# Mineral Acids in Nausea, and Vomiting in Pregnancy

**Published:** 1855-05

**Authors:** 


### MINERAL ACIDS IN NAUSEA AND VOMITING DURING PREGNANCY.

Of the many diseases incident to the state of pregnancy in the human female, no one is more troublesome or a greater source of annoyance to the patient, friends, and physician, than nausea and its sequent, vomiting.

Not only does it manifest itself in the early stage of pregnancy but continues even to the end, the last months being often more severe than the first, and in some cases does not manifest itself until the 8th or 9th month.

That its cause during the first stage of fœtal existence is dependent upon and due to the sympathetic action of the gestative organ upon the stomach is evident, especially when we see that derangement of the digestive functions is a consequent disease of the uterus from any cause whatever.

When it occurs during the latter stages, mechanical pressure or an over fullness of the genital system have been considered the exciting causes, and in many cases, if not in all, will account for the disease. Torpidity of the bowels and the ingestion of indigestible food will also tend to produce it.

To relieve and if possible, to obviate this state of things, necessarily devolves upon the physician ; and how often do the best directed efforts fail to bring about the desired results ? My attention has been directed to this subject from several patients coming under my charge afflicted with this disease.

In the spring of 1853, I was called to see Mrs. G., who was said to be nearly dead from long-continued vomiting. She was pregnant for the first time, and was then about eight months gone.

Deeming, upon examination, that the cause of the vomiting was mechanical pressure, I could promise her no relief, until it should take place by her confinement ; but as she was suffering so much, and as no food, from the richest to the most bland, would remain in the stomach, I was induced to try what could be done for her. Each in its turn, I prescribed the usual remedies with little, and sometimes with no benefit, and was about giving the case up as irremediable when I thought of the mineral acids ; and making a weak solution of sulphuric acid and water, I gave it to her, with directions to use it every hour. She did so, and the first dose, in a measure, relieved her, and after a few doses she was enabled to retain some food. She was troubled but little afterwards, when, her confinement taking place, she entirely recovered. In August, of the present year, she was again in a similar situation as above related, when I treated her as before, but with no relief of the symptoms, till I administered the acids. This time I gave the nitric acid, diluted as above, with the same good results.

In October last, I was called to see Mrs. M., who, her husband informed me, could not live long unless something was speedily done for her, as she had been vomiting for nearly two months, and at the time was suffering from diarrhœa.

I found the woman emaciated to almost a skeleton. She was confined to her bed and unable to sit up, and could retain nothing whatever in the stomach. She was then, as she thought, about two months pregnant ; and had not consulted a physician before because her neighbors had told her all women were so troubled ; and being her first pregnancy, she felt a delicacy in so doing.

I prescribed for her the dilute sulphuric acid, to which I added some morphine, to relieve the pain of the diarrhœa. She felt relieved after the first dose, and in a week was able to sit up, and soon entirely recovered. I have used it in several other cases, as have some of my medical friends, with the same beneficial results ; nor have I as yet been disappointed.—*Peninsular Jour. Med.*

